# Racial inequalities in multimorbidity: baseline of the Brazilian Longitudinal Study of Adult Health (ELSA-Brasil)

**DOI:** 10.1186/s12889-022-13715-7

**Published:** 2022-07-09

**Authors:** Fernanda Esthefane Garrides Oliveira, Rosane Harter Griep, Dora Chor, Luana Giatti, Luciana A. C. Machado, Sandhi Maria Barreto, Alexandre da Costa Pereira, Maria de Jesus Mendes da Fonseca, Leonardo Soares Bastos

**Affiliations:** 1 Sérgio Arouca National School of Public Health, Oswaldo Cruz Foundation, Rio de Janeiro, Brazil; 2 Laboratory of Health and Environment Education, Oswaldo Cruz Institute, Rio de Janeiro, Brazil; 3 Department of Preventive and Social Medicine, Federal University of Minas Gerais, Belo Horizonte, Brazil; 4 Clinical Hospital/EBSERH, Federal University of Minas Gerais, Belo Horizonte, Brazil; 5 Heart Institute, University of São Paulo, São Paulo, Brazil; 6 Scientific Computing Program, Oswaldo Cruz Foundation, Rio de Janeiro, Brazil

**Keywords:** Multimorbidity, Chronic disease, Disease accumulation, Racial inequalities in health, Social determinants of health

## Abstract

**Background:**

Evidence of multimorbidity has come mainly from high-income regions, while disparities among racial groups have been less explored. This study examined racial differences in multimorbidity in the multiracial cohort of the Longitudinal Study of Adult Health (*Estudo Longitudinal de Saúde do Adulto*), ELSA-Brasil.

**Methods:**

The study examined baseline (2008–2010) data for 14 099 ELSA-Brasil participants who self-reported being white, mixed-race, or black. A list of 16 morbidities was used to evaluate multimorbidity, operationalised by simple count into ≥ 2, ≥ 3, ≥ 4, ≥ 5 and ≥ 6 morbidities, in addition to evaluating the number of coexisting conditions. Prevalence ratios (PR) were estimated from logistic models and a quantile model was used to examine racial differences graphically in the distribution quantiles for the number of morbidities.

**Results:**

Overall prevalence of multimorbidity (≥ 2 morbidities) was 70% and, after controlling for age and sex, was greater among mixed-race and black participants – by 6% (PR: 1.06; 95% CI: 1.03–1.08) and 9% (PR: 1.09; 95% CI: 1.06–1.12), respectively – than among white participants. As the cutoff value for defining multimorbidity was raised, so the strength of the association increased, especially among blacks: if set at ≥ 6 morbidities, the prevalence was 27% greater for those of mixed-race (PR: 1.27; 95% CI: 1.07–1.50) and 47% greater for blacks (PR: 1.47; 95% CI: 1.22–1.76) than for whites. The disparities were smaller in the lower morbidity distribution quantiles and larger in the upper quantiles, indicating a heavier burden of disease, particularly on blacks.

**Conclusions:**

Multimorbidity was common among adults and older adults in a Brazilian cohort, but important racial inequalities were found. Raising the cutoff point for defining multimorbidity revealed stronger associations between race/skin colour and multimorbidity, indicating a higher prevalence of multimorbidity among mixed-race and black individuals than among whites and that the former groups coexisted more often with more complex health situations (with more coexisting morbidities). Interventions to prevent and manage the condition of multimorbidity that consider the social determinants of health and historically discriminated populations in low- and middle-income regions are necessary.

**Supplementary Information:**

The online version contains supplementary material available at 10.1186/s12889-022-13715-7.

## Background

The rapid population aging seen globally in recent decades has been accompanied by increasing prevalence of long-term conditions and related deaths [1, 2]. In this scenario, multimorbidity, two or more chronic conditions coexisting in the same individual [1], becomes frequent and challenges economies and health systems the world over to guarantee aging with quality of life, with preventive measures and equitable health care and patient safety, in a context of complex therapy plans involving a diversity of medicines and fragmented care [1, 3, 4].

Epidemiological evidence of multimorbidity has come mainly from high-income countries [3], where it is considered the norm rather than an exception [4], but populations of other regions are gradually living with multimorbidity [5]. Community-based studies in adults have found prevalences between 3.51% and 70.14% for high-income regions and between 0.66% and 90.47% for low- and middle-income regions [6]. Estimates vary widely due to the heterogeneity of samples, the nature and number of conditions assessed and how multimorbidity is operationalised [3, 7]. They nonetheless converge in stressing that multimorbidity is an important health phenomenon which affects not only older populations in high-income regions [5–7].

There is a direct relation between advancing age and multimorbidity [6], but middle-aged adults are also experiencing this situation [6, 8] and unequally so between population subgroups. Higher prevalences are reported for women [5, 6] and certain racial groups [9], such as blacks in the United States of America (USA), who reach situations of multimorbidity younger than whites and live with more morbidities over time [10, 11].

Living with multimorbidity is associated with a variety of outcomes, including catastrophic health expenditures [12], high levels of service use, in both instances of ambulatory care and days of hospital stay [3, 12, 13], polypharmacy, loss of functional capacity, worse quality of life and greater risk of death [6, 13, 14]. Some of these relations, such as greater likelihood of polypharmacy and worsening of functional decline [15, 16], grow stronger the number of coexisting morbidities increases, as when multimorbidity is defined in ≥ 3 or ≥ 4 morbidities. In addition, more severe or complex multimorbidity – coexistence of three or more morbidities affecting three or more different body systems [17] – has been associated with greater limitation in activities of daily living [18, 19]. Based on these indications, raising the cutoff point to define the situation of multimorbidity may help identify individuals or groups that require differentiated assistance and are at greater risk of clinical worsening.

In low- and middle-income countries, the impact of multimorbidity can be even more marked, due to an unfinished agenda of transitions [4], with infectious and chronic diseases sharing the onus of morbi-mortality with external causes [20] in contexts of extreme social inequalities, fragile health systems and worsening risk factors, such as unplanned urbanisation, sedentary lifestyles, and unhealthy eating patterns [4, 8, 20].

In Brazil, a middle-income economy with a miscegenated population historically marked by racial inequalities [21], most of the evidence about multimorbidity has been published in the past five years using data from the National Heath Survey [22] and for adults at least 45 years old [23]. The study of related social determinants is still incipient and without prioritizing disparities among racial groups. To address that gap, this study examined the association between race/skin colour and prevalence of multimorbidity, at different cutoff points, and evaluated differences in numbers of morbidities between racial groups at the baseline of the Brazilian Longitudinal Study of Adult Health (*Estudo Longitudinal de Saúde do Adulto*), ELSA-Brasil.

## Methods

### Study design and participants

ELSA-Brasil is a multicenter study of a prospective cohort of 15 105 active and retired civil servants between 35 and 74 years old from six higher education and/or research institutions in Brazilian state capitals in three of the five geographical regions of the country: Southeast (Belo Horizonte, Rio de Janeiro, São Paulo and Vitória), South (Porto Alegre) and Northeast (Salvador) [24]. ELSA’s main objectives are to investigate the development and progression of chronic diseases and their determinants [25]. Baseline data on the cohort were collected in person between 2008 and 2010 in interviews based on previously tested questionnaires and clinical, laboratory and imaging exams. Information on the methodology and cohort profile was published previously [24–26].

This study is a cross-sectional analysis that includes 14 099 (93.34%) participants from the ELSA-Brasil baseline, following exclusions for missing data (*n* = 475) and those of self-reported Asian (*n* = 374) or indigenous descent (*n* = 157), given the low frequency and unfeasibility of pooling these groups (Additional File 1 shows a flow diagram of the exclusion process).

## Measures

### Multimorbidity

Multimorbidity was assessed using a list of 16 chronic morbidities, ten of which were self-reported in response to the question *Has a doctor ever informed you that you had or have (…)?*: cancer; rheumatic fever; ischemic heart disease (angina and/or myocardial infarction); cardiac insufficiency; cerebrovascular accident; emphysema, chronic bronchitis or chronic obstructive pulmonary disease (COPD); asthma; liver cirrhosis or hepatitis; joint disorders; or renal disease.

The other six morbidities were assessed by a series of data. Diabetes was specified as a self-reported diagnosis and/or use of medicines and/or by laboratory data for fasting glucose (≥ 126 mg/dL), glycated haemoglobin (≥ 6.5%) and 2-h 75 g glucose tolerance test (≥ 200 mg/dL). Dyslipidaemia was specified by low density lipoprotein cholesterol levels after 12-h fasting (≥ 130 mg/dL) or use of hypolipidemic. Obesity was specified by body mass index (≥ 30 kg/m^2^), based on anthropometric measurements taken following the study protocols [26]. Hypertension was specified as systolic arterial pressure (≥ 140 mmHg) and/or diastolic arterial pressure (≥ 90 mmHg) and/or use of an anti-hypertensive. Arterial pressure is the mean of the two last measurements of a series of three taken at one-minute intervals with an oscillometric device, while resting in a controlled environment [26]. Migraine was specified by the diagnostic criteria of International Headache Society codes 1.1 (without aura), 1.2 (with aura) and 1.6 (maybe) [27], as assessed by a headache questionnaire translated into Brazilian Portuguese and used previously [28].

The presence of common non-psychotic mental disorders was assessed by the Clinical Interview Schedule – Revised Version (CIS-R), as translated and adapted for the ELSA-Brasil population [29]. The CIS-R is composed of 14 sections that assess the presence and severity of psychological symptoms: somatic symptoms, fatigue, concentration/memory problems, sleep problems, irritability, preoccupation with physical symptoms, depression, depressive ideas, worries, anxiety, phobias, panic, compulsions, and obsessions [30]. Two screening questions in each section ask about the presence of the symptom during the last month and, if so, there is a more detailed assessment of the presence, frequency, intensity, duration, and degree of bother caused by the symptom during the last seven days. Scores can range from zero to four in each section, except for the section on depressive ideas which ranges from zero to five. A CIS-R total score ≥ 12 was used to define cases with any common mental disorder [30–32] and is the definition adopted in ELSA-Brasil.

Multimorbidity status was specified from a simple count of the 16 morbidities and was used both in that form and categorised at five cutoff points: ≥ 2, ≥ 3, ≥ 4, ≥ 5 and ≥ 6 morbidities. No tests were performed at higher cutoff points because the prevalence of multimorbidity in such cases is less than 5%.

### Race/skin colour

In ELSA-Brasil, race/skin colour was self-classified by the options used by Brazil’s official bureau of statistics (*Instituto Brasileiro de Geografia e Estatística* – IBGE) with the question: *The Brazilian population census uses the terms black, mixed-race, white, yellow (Asian descent), and indigenous to classify people by colour or race. If you had to respond to the census today, how would you classify yourself by colour or race?* Mixed-race participants and blacks were compared with whites, with race/skin colour being understood as a sociocultural construct [21], a risk marker potentially able to reveal discriminatory processes and proxy for the lived experience and oppressive social relations that place some population groups at a life course disadvantage [33].

### Covariates

Age (in years) and sex (male or female) were included to adjust the models, and age groups in the graphical analyses. According to the available literature [21, 33–35], racism and racial discrimination influence socioeconomic position, health care and health risk behaviour – which are part of the pathway connecting race/skin colour with health outcomes. Accordingly, six factors related to socioeconomic position and health risk behaviours were considered only to describe participants and in sensitivity analyses.

Education levels are categorised as complete higher education, complete high school, complete elementary school, and up to incomplete elementary school. The *per capita* family income is categorised into quintiles, dividing into five equal parts the values estimated from the information reported by the participants on monthly family income and the number of dependants of this income, thus the first quintile corresponds to ≤ R$622.42 (about ≤ U$311. 58 by the 2009 average exchange rate) and the fifth quintile corresponds to > R$2628.17 and ≤ R$7884.50 (about > U$1315.66 and ≤ U$3946.99 by the 2009 average exchange rate). Owning or not owning a health insurance plan is defined by the response to the question: *Do you have a private plan for health care?* Smoking is categorised as a non-smoker, former smoker, and current smoker. The alcohol consumption is assessed by a questionnaire on the consumption of types of alcoholic beverage and frequency of drinking, then the alcohol content and amount consumed are estimated and hazardous drinking is defined as present if ≥ 210 g of ethanol for men and ≥ 140 g for women, according to health risk limits defined in several countries [36]. And leisure-time physical activity is categorised as weak if < 600 min/week, moderate if 600–3000 min/week and vigorous if ≥ 3000 min/week, assessed by the long version of the International Physical Activity Questionnaire [37].

### Statistical analysis

The cohort baseline was described by racial group (and by multimorbidity status, in the Additional File 2), using absolute frequencies and proportions, with Pearson’s chi-square test for the categorical variables, and means, standard deviations, medians, 1^st^ and 3^rd^ quartile and Kruskal–Wallis test for age and number of morbidities. Graphs were used to describe the prevalence of multimorbidity (and each morbidity, in the Additional File 3) among the racial groups, by sex and specific age groups, and for the distribution of number of morbidities among the racial groups.

The association between self-reported race/skin colour and multimorbidity was evaluated by logistic model, using a direct approach to estimate prevalence ratios (PRs) obtained by a conditional method [38, 39] and respective 95% confidence intervals (95% CI). Adjustments were made for confounders (sex and age), the Akaike Information Criterion (AIC) was reported to the models and the Hosmer–Lemeshow test of goodness of fit was used in the final models.

The quantile model [40, 41] was used to describe the association between race/skin colour and number of morbidities. This model was chosen because the tail of the distribution of the number of morbidities was heavier towards mixed-race and black participants, and outliers were found in the three racial groups. Accordingly, it was decided not to model for the mean number of morbidities. The advantage of the quantile model over the logistic model is that it enables extreme outcome situations to be assessed, without categorisation, and provides more information on the associations [41]. Conditioning the model to age and sex, coefficients were estimated between the 5^th^ and 95^th^ quantiles of the outcome distribution. The result was displayed graphically, and the coefficients express the difference in the number of morbidities for mixed-race and black participants as compared with white participants.

Sensitivity analyses were performed on the models that estimated PRs. First, to evaluate the potential contribution of including socioeconomic factors (schooling, family income and health insurance) and health risk behaviour (smoking, excessive weekly alcohol consumption and leisure-time physical inactivity) in the age- and sex-adjusted models (Additional File 4). In another analysis (Additional File 5), the risk marker of interest was altered to a variable acting as proxy for the intersection among social identities (white men, mixed-race men, black men, white women, mixed-race women, and black women). Subsequently, the association of interest was explored when the outcome was specified by a list of 13 morbidities (Additional File 6), i.e., excluding those that could be considered risk factors and were among the most prevalent (> 20%) among ELSA participants (dyslipidaemia, arterial hypertension, and obesity). Lastly, the association of interest was explored when the outcome was specified by a list of six morbidities, considering only those measured in ELSA-Brasil by clinical or laboratory tests or diagnostic questionnaires: dyslipidaemia, arterial hypertension, migraine, common mental disorders, obesity, and diabetes (Additional File 7).

All analyses were performed using the R statistical software (version 4.0.3), and a significance level of 5% was considered.

## Results

Of the total of 14 099 participants, 53.99% self-reported their race/skin colour to be white (n = 7 612), 29.25% mixed-race (*n* = 4 124) and 16.76% black (n = 2 363). Median age was 51 years, and most participants (54.32%) were female (Table 1). The distribution by age group was close between the racial groups, but the white group had proportionally more adults aged 60 and over. The white group also has a higher proportion of participants with complete higher education, in the highest quintiles of *per capita* family income, and with health insurance plans. While current smokers, hazardous drinkers and those who practise less physical activity were more frequent among mixed-race and black participants (Table 1). The descriptive analysis by a cutoff of multimorbidity showed that the higher this cutoff, the more frequent were women, participants of lower educational level, lower family income, weak physical activity, and higher median age in the group with multimorbidity (Additional File 2).

**Table 1:** Descriptive characteristics of participants, ELSA-Brasil baseline

Baseline characteristics^a^	Overall (%)^b^	Race/skin colour (%)^b^			*p* Value^c^
		**White**	**Mixed-race**	**Black**	
**Total number of participants**	14 099 (100)	7612 (53.99)	4124 (29.25)	2363 (16.76)	
**Demographic**					
*Age in years (n* = *14 099)*					
Mean (standard deviation)	51.98 (9.06)	52.48 (9.36)	51.16 (8.65)	51.81 (8.67)	< 0.001
Median (1^st^ quartile—3^rd^ quartile)	51 (45—58)	52 (45—59)	51 (45—57)	51 (45—58)	
*Age groups (n* = *14 099)*					
35–39 years	1097 (7.78)	583 (7.66)	361 (8.75)	153 (6.47)	< 0.001
40–44 years	2052 (14.55)	1091 (14.33)	603 (14.62)	358 (15.15)	
45–49 years	2949 (20.92)	1498 (19.68)	933 (22.62)	518 (21.92)	
50–54 years	2644 (18.75)	1347 (17.70)	827 (20.05)	470 (19.89)	
55–59 years	2376 (16.85)	1290 (16.95)	685 (16.61)	401 (16.97)	
60–64 years	1532 (10.87)	893 (11.73)	385 (9.34)	254 (10.75)	
65–69 years	886 (6.28)	536 (7.04)	221 (5.36)	129 (5.46)	
70 + years	563 (3.99)	374 (4.91)	109 (2.64)	80 (3.39)	
*Sex (n* = *14 099)*					
Male	6441 (45.68)	3520 (46.24)	1995 (48.38)	926 (39.19)	< 0.001
Female	7658 (54.32)	4092 (53.76)	2129 (51.62)	1437 (60.81)	
**Socioeconomic position**					
*Education levels (n* = *14 099)*					
Complete higher education	7381 (52.35)	5091 (66.88)	1653 (40.08)	637 (26.96)	< 0.001
Complete high school	4949 (35.10)	1974 (25.93)	1769 (42.90)	1206 (51.04)	
Complete elementary school	962 (6.82)	310 (4.07)	361 (8.75)	291 (12.31)	
Up to incomplete elementary school	807 (5.72)	237 (3.11)	341 (8.27)	229 (9.69)	
*Per capita family income (n* = *14 059)*					
5^th^ (> US$ 1315.66 and ≤ US$ 3946.99)	2278 (16.20)	1747 (23.00)	375 (9.12)	156 (6.63)	< 0.001
4^th^ (> US$ 882.88 and ≤ US$ 1315.66)	3176 (22.59)	2213 (29.14)	686 (16.69)	277 (11.77)	
3^rd^ (> US$ 519.25 and ≤ US$ 882.88)	2907 (20.68)	1675 (22.05)	849 (20.65)	383 (16.28)	
2^nd^ (> US$ 311.58 and ≤ US$ 519.25)	2860 (20.34)	1143 (15.05)	1059 (25.76)	658 (27.96)	
1^st^ (≤ US$ 311.58)	2838 (20.19)	817 (10.76)	1142 (27.78)	879 (37.36)	
*Health insurance plans (n* = *14 098)*					
Yes	9629 (68.30)	5539 (72.78)	2721 (65.98)	1369 (57.93)	< 0.001
No	4469 (31.70)	2072 (27.22)	1403 (34.02)	994 (42.07)	
**Behaviours**					
*Smoking (n* = *14 098)*					
Non-smoker	8044 (57.06)	4295 (56.42)	2374 (57.58)	1375 (58.19)	< 0.001
Former smoker	4214 (29.89)	2388 (31.37)	1184 (28.72)	642 (27.17)	
Current smoker	1840 (13.05)	929 (12.20)	565 (13.70)	346 (14.64)	
*Hazardous drinking (n* = *14 090)*					
No	13,032 (92.49)	7074 (92.98)	3785 (91.82)	2173 (92.05)	0.054
Yes	1058 (7.51)	534 (7.02)	337 (8.18)	187 (7.92)	
*Physical activity (n* = *13 899)*					
Weak	10,680 (76.84)	5524 (73.82)	3220 (79.10)	1936 (82.56)	< 0.001
Moderate	2232 (16.06)	1362 (18.20)	579 (14.22)	291 (12.41)	
Vigorous	987 (7.10)	597 (7.98)	272 (6.68)	118 (5.03)	

^a^The total number of participants with complete information for each of the characteristics is indicated in parentheses

^b^Percentages within each race/skin colour group or overall estimate (sum 100% in the column), except for the numerical variable age for which mean, standard deviation, median, 1^st^ and 3^rd^ quartile are given; and the total number of participants that adds up to 100% on the line

^c^Refers to the X^2^ test for difference in percentages and Kruskal–Wallis test for age between race/skin colour groups

The most prevalent morbidities (Table 2) were dyslipidaemia (45.83%), which was most common in whites (reaching 47.19% of this group), followed by arterial hypertension (35.87%), most common in blacks (reaching 48.67% of this group). Blacks were found to display higher prevalences of migraine, common mental disorders, obesity, joint disorders, diabetes, ischemic heart disease, and cardiac insufficiency. Whites returned higher prevalences of renal disease, liver cirrhosis or hepatitis, cancer and emphysema, chronic bronchitis, or COPD (Table 2). The prevalence of each morbidity among racial groups by sex and age group detailed these differences (Additional File 3): arterial hypertension and diabetes were more prevalent in blacks than in whites, but mainly between 50 and 64 years, both for males and females; from 40 to 64 years old, the prevalence of common mental disorders was higher among female participants self-classified as mixed-race and black; and aged 50 to 69 years, cancer was more prevalent among whites than among black females.

**Table 2:** Prevalence of morbidities and of multimorbidity at different cutoffs, ELSA-Brasil baseline

Baseline characteristics^a^	Overall (%)^b^	Race/skin colour (%)^b^			*p* Value^c^
		**White**	**Mixed-race**	**Black**	
**Morbidities**					
*Dyslipidaemia*					
No	7637 (54.17)	4020 (52.81)	2291 (55.55)	1326 (56.12)	0.002
Yes	6462 (45.83)	3592 (47.19)	1833 (44.45)	1037 (43.88)	
*Arterial hypertension*					
No	9042 (64.13)	5238 (68.81)	2591 (62.83)	1213 (51.33)	< 0.001
Yes	5057 (35.87)	2374 (31.19)	1533 (37.17)	1150 (48.67)	
*Migraine*					
No	9921 (70.37)	5425 (71.27)	2877 (69.76)	1619 (68.51)	0.023
Yes	4178 (29.63)	2187 (28.73)	1247 (30.24)	744 (31.49)	
*Common mental disorders*					
No	10 320 (73.20)	5845 (76.79)	2861 (69.37)	1614 (68.30)	< 0.001
Yes	3779 (26.80)	1767 (23.31)	1263 (30.63)	749 (31.70)	
*Obesity*					
No	10 863 (77.05)	6025 (79.15)	3182 (77.16)	1656 (70.08)	< 0.001
Yes	3236 (22.95)	1587 (20.85)	942 (22.84)	707 (29.92)	
*Joint disorders*					
No	11 065 (78.48)	6092 (80.03)	3197 (77.52)	1776 (75.16)	< 0.001
Yes	3034 (21.52)	1520 (19.97)	927 (22.48)	587 (24.84)	
*Renal disease*					
No	11 616 (82.39)	6041 (79.36)	3469 (84.12)	2106 (89.12)	< 0.001
Yes	2483 (17.61)	1571 (20.64)	655 (15.88)	257 (10.88)	
*Diabetes*					
No	11 866 (84.16)	6602 (86.73)	3445 (83.54)	1819 (76.98)	< 0.001
Yes	2233 (15.84)	1010 (13.27)	679 (16.46)	544 (23.02)	
*Asthma*					
No	12 457 (88.35)	6696 (87.97)	3674 (89.09)	2087 (88.32)	0.194
Yes	1642 (11.65)	916 (12.03)	450 (10.91)	276 (11.68)	
*Liver cirrhosis or hepatitis*					
No	12 886 (91.40)	6817 (89.56)	3843 (93.19)	2226 (94.20)	< 0.001
Yes	1213 (8.60)	795 (10.44)	281 (6.81)	137 (5.80)	
*Cancer*					
No	13 449 (95.39)	7151 (93.94)	3996 (96.90)	2302 (97.42)	< 0.001
Yes	650 (4.61)	461 (6.06)	128 (3.10)	61 (2.58)	
*Ischemic heart disease*					
No	13 482 (95.62)	7297 (95.86)	3949 (95.76)	2236 (94.63)	0.033
Yes	617 (4.38)	315 (4.14)	175 (4.24)	127 (5.37)	
*Rheumatic fever*					
No	13 697 (97.15)	7389 (97.07)	4003 (97.07)	2305 (97.55)	0.446
Yes	402 (2.85)	223 (2.93)	121 (2.93)	58 (2.45)	
*Emphysema, chronic bronchitis or COPD*					
No	13 819 (98.01)	7426 (97.56)	4063 (98.52)	2330 (98.60)	< 0.001
Yes	280 (1.99)	186 (2.44)	61 (1.48)	33 (1.40)	
*Cardiac insufficiency*					
No	13 871 (98.38)	7519 (98.78)	4051 (98.23)	2301 (97.38)	< 0.001
Yes	228 (1.62)	93 (1.22)	73 (1.77)	62 (2.62)	
*Cerebrovascular accident*					
No	13 920 (98.01)	7526 (98.87)	4068 (98.52)	2326 (98.43)	0.213
Yes	179 (1.27)	86 (1.13)	61 (1.48)	37 (1.57)	
**Multimorbidity cutoff**					
≥ *2 morbidities*					
No	4217 (29.91)	2409 (31.65)	1210 (29.34)	598 (25.31)	< 0.001
Yes	9882 (70.09)	5203 (68.35)	2914 (70.66)	1765 (74.69)	
≥ *3 morbidities*					
No	7620 (54.05)	4254 (55.89)	2233 (54.15)	1133 (47.95)	< 0.001
Yes	6479 (45.95)	3358 (44.11)	1891 (45.85)	1230 (52.05)	
≥ *4 morbidities*					
No	10 484 (74.36)	5789 (76.05)	3075 (74.56)	1620 (68.56)	< 0.001
Yes	3615 (25.64)	1823 (23.95)	1049 (25.44)	743 (31.44)	
≥ *5 morbidities*					
No	12 360 (87.67)	6742 (88.57)	3626 (87.92)	1992 (84.30)	< 0.001
Yes	1739 (12.33)	870 (11.43)	498 (12.08)	371 (15.70)	
≥ *6 morbidities*					
No	13 359 (94.75)	7255 (95.31)	3902 (94.62)	2202 (93.19)	< 0.001
Yes	740 (5.25)	357 (4.69)	222 (5.38)	161 (6.81)	
*Number of morbidities*					
Mean (standard deviation)	2.53 (1.69)	2.45 (1.65)	2.53 (1.69)	2.78 (1.78)	< 0.001
Median (1^st^—3^rd^ quartile)	2 (1—4)	2 (1—3)	2 (1—4)	3 (1—4)	

*COPD* chronic obstructive pulmonary disease

^a^N for all characteristics was 14 099

^b^Percentages within each race/skin colour group or overall estimate (sum 100% in the column), except for the numerical variable age for which mean, standard deviation, median, 1^st^ and 3^rd^ quartile are given and the total number of participants that adds up to 100% on the line

^c^Refers to the X^2^ test for difference in percentages and Kruskal–Wallis test for number of morbidities between race/skin colour groups

The overall prevalence of multimorbidity (Table 2) was 70.09% on the classic definition (≥ 2 morbidities), but 68.35% among whites, 70.66% among mixed-race and 74.69% among blacks. As the cutoff point used to specify multimorbidity was raised, so the overall prevalence diminished to 5.25% (≥ 6 morbidities), i.e., 4.69%, 5.38% and 6.81% among white, mixed-race, and black participants, respectively. Regardless of how multimorbidity was operationalised, black participants returned higher prevalences, although the higher the cutoff point, the more the estimates approached each other.

**Fig. 1:**
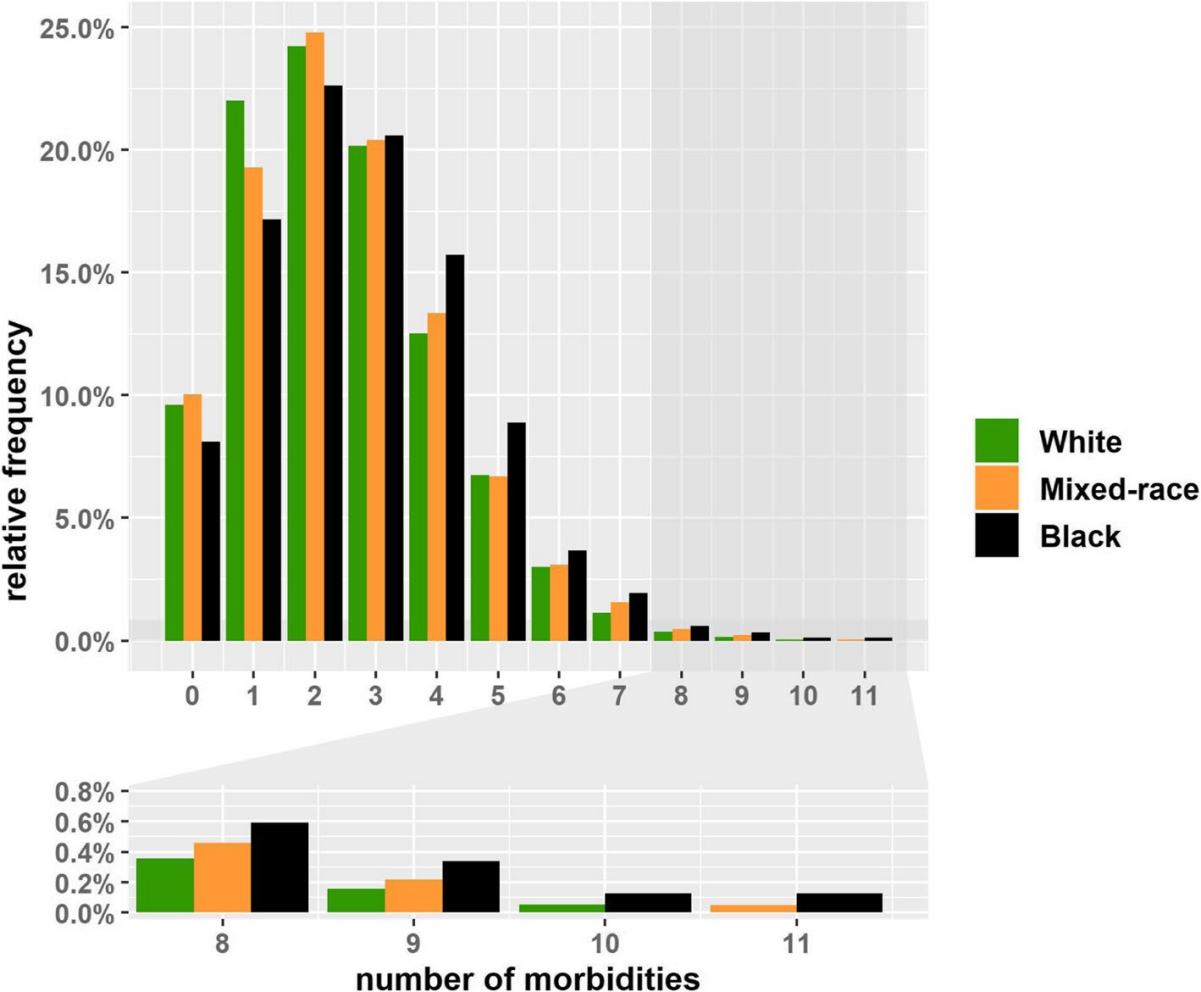
Frequency distribution of number of coexisting morbidities by race/skin colour group, ELSA-Brasil baseline. Notes: the bottom graph is an amplified image of categories of eight to eleven morbidities

On average, white participants lived with 2.45 morbidities, mixed-race individuals with 2.53 and blacks with 2.78 (Table 2). Among whites, 75% lived with none or up to three morbidities (3^rd^ quartile: 3), while 50% of blacks lived with three or more (median: 3). Figure 1 illustrates these differences: most of the whites were free from morbidities or presented up to two, while those of mixed-race and blacks lived with more morbidities, comprised higher percentages of individuals with seven up to eleven of the 16 morbidities considered in this study.

**Fig. 2:**
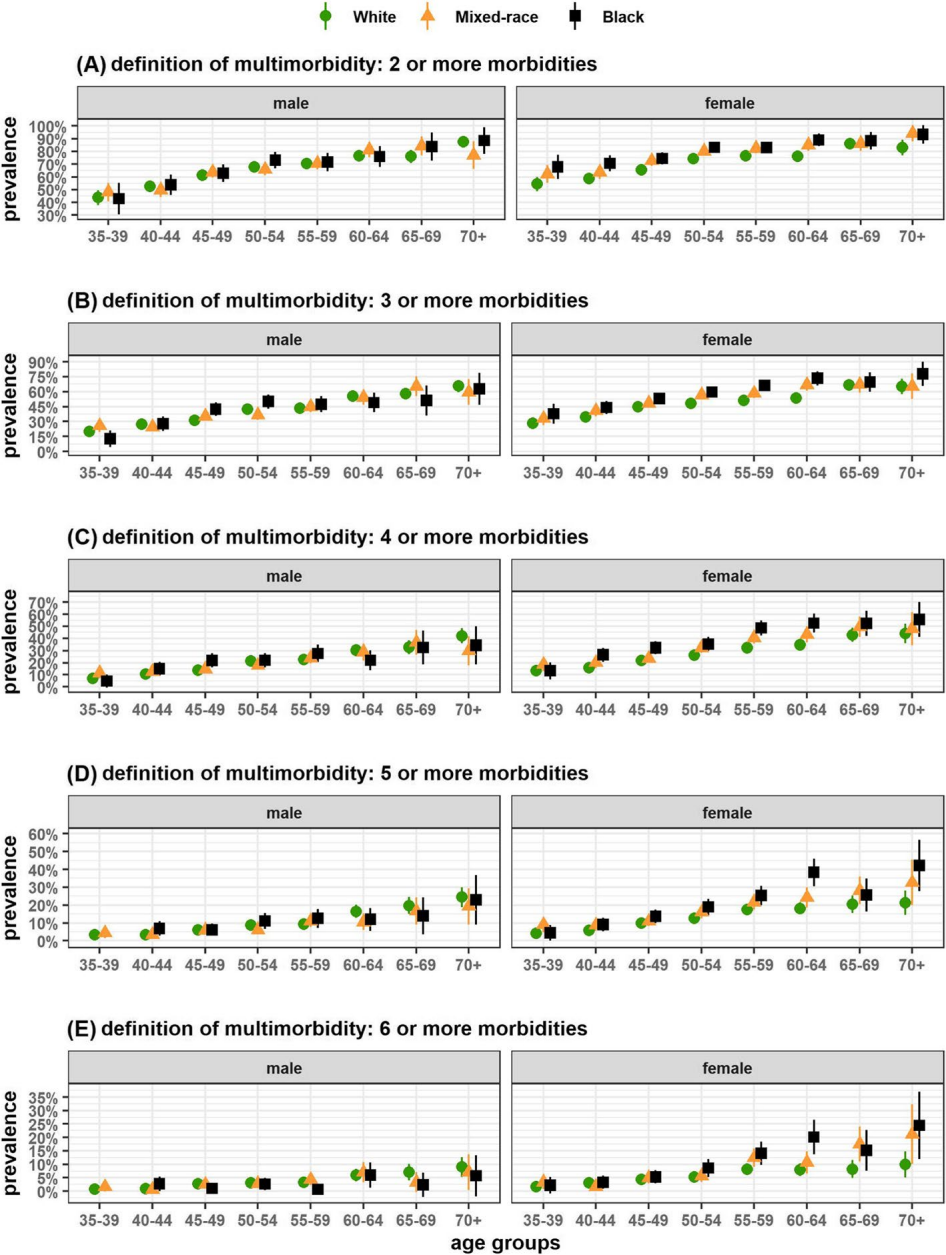
Prevalence of multimorbidity at different cutoffs in specific age groups and sex, ELSA-Brasil baseline. Notes: vertical lines correspond to the 95% confidence interval of the prevalence estimates

The prevalences of multimorbidity among the racial groups by sex and age groups (Fig. 2) revealed that, on any operational definition of multimorbidity, black and mixed-race female participants of more advanced age presented higher prevalences. Prevalences among black and white females differed in the age groups from 40 to 64 years when multimorbidity was specified as ≥ 2, ≥ 3 and ≥ 4 morbidities (Fig. 2-A, 2-B, and 2-C respectively); when specified at higher cutoffs, the differences resided in the 55 to 64 age groups (Fig. 2-D and 2-E). Differences among male participants were slighter, although there were indications that, in the youngest age group, mixed-race showed higher prevalence and, in the most advanced age group, multimorbidity was more common in white.

Table 3 shows PRs for each situation where the specification of multimorbidity was altered. In all cases of unadjusted estimates, blacks showed higher prevalences of multimorbidity than whites, but only on the classic definition (≥ 2 morbidities) did mixed-race participants show higher prevalence than whites. Adjusting for sex (Model 1) attenuated the estimates for blacks and the prevalence for mixed-race became higher than that of whites up to the cutoff point of ≥ 4 morbidities. Age acted as a negative confounder (Model 2): when not controlled for, led to underestimation of the strength of the association. Lastly, the model adjusted for age and sex (Final Model) revealed that the higher the cutoff point specifying multimorbidity, the stronger the association for both mixed-race individuals and blacks. Prevalence was greater in mixed-race participants by from 6% (PR: 1.06; 95% CI: 1.03–1.08) to 27% (PR: 1.27; 95% CI: 1.07–1.50) and, in blacks, by from 9% (PR: 1.09; 95% CI: 1.06–1.12) to 47% (PR: 1.47; 95% CI: 1.22–1.76), both as compared with whites. The test to assess the loss of goodness of fit of the final models indicated that there was no loss in any of the models with different definitions of multimorbidity. The best AIC was found in the operationalisation of multimorbidity as ≥ 6 morbidities.

**Table 3:** Models for the association between race/skin colour and multimorbidity at different cutoffs, ELSA-Brasil baseline

Multimorbidity cutoff^a^	Crude PR (95% CI)	Model 1^b^	Model 2^c^	Final Model^d^	
		**PR (95% CI)**	**PR (95% CI)**	**PR (95% CI)**	***p* Value**^**e**^
** ≥ 2 morbidities**					
Mixed-race	1.03 (1.01–1.06)**	1.04 (1.01–1.06)**	1.05 (1.03–1.08)***	1.06 (1.03–1.08)***	0.722
Black	1.09 (1.06–1.12)***	1.08 (1.05–1.11)***	1.10 (1.07–1.13)***	1.09 (1.06–1.12)***	
*AIC*	*17 173*	*17 068*	*16 566*	*16 454*	
** ≥ 3 morbidities**					
Mixed-race	1.04 (1.00–1.08)	1.04 (1.002–1.09)*	1.08 (1.04–1.12)***	1.09 (1.04–1.13)***	0.282
Black	1.18 (1.13–1.23)***	1.16 (1.11–1.22)***	1.21 (1.15–1.26)***	1.19 (1.14–1.25)***	
*AIC*	*19 413*	*19 264*	*18 734*	*18 572*	
** ≥ 4 morbidities**					
Mixed-race	1.06 (1.00–1.13)	1.07 (1.003–1.14)*	1.13 (1.06–1.21)***	1.14 (1.06–1.22)***	0.223
Black	1.31 (1.22–1.41)***	1.28 (1.20–1.38)***	1.37 (1.28–1.48)***	1.34 (1.25–1.44)***	
*AIC*	*16 006*	*15 836*	*15 417*	*15 231*	
** ≥ 5 morbidities**					
Mixed-race	1.06 (0.95–1.17)	1.07 (0.96–1.18)	1.15 (1.04–1.28)**	1.16 (1.04–1.28)**	0.132
Black	1.37 (1.23–1.54)***	1.33 (1.19–1.49)***	1.46 (1.30–1.64)***	1.41 (1.26–1.59)***	
*AIC*	*10 510*	*10 401*	*10 079*	*9960*	
** ≥ 6 morbidities**					
Mixed-race	1.15 (0.98–1.35)	1.17 (0.99–1.37)	1.27 (1.07–1.49)**	1.27 (1.07–1.50)**	0.921
Black	1.45 (1.21–1.74)***	1.39 (1.16–1.67)***	1.55 (1.29–1.86)***	1.47 (1.22–1.76)***	
*AIC*	*5793*	*5696*	*5580*	*5477*	

*PR* prevalence ratios, *95% CI* 95% confidence interval, *AIC* Akaike Information Criterion

^a^Reference category in all models: race/skin colour white

^b^Model 1: adjusted for sex

^c^Model 2: adjusted for age

^d^Final Model: adjusted for sex and age

^e^*p* value for Hosmer–Lemeshow test for loss of quality in Final Model fit

Significance: *** *p* value ≤ 0.001; ** 0.001 < *p* value ≤ 0.01; * 0.01 < *p* value < 0.05

**Fig. 3:**
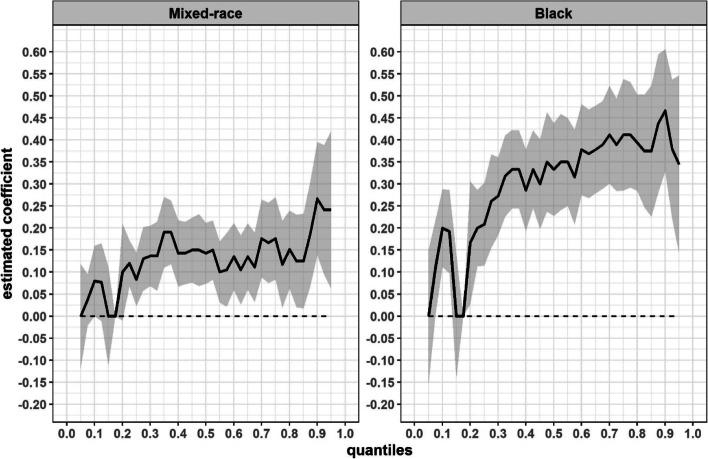
Quantile regression for the association between race/skin colour and number of coexisting morbidities, ELSA-Brasil baseline. Notes: white race/skin colour as reference and the model was adjusted for age and sex. The solid black line indicates the coefficient estimated in each quantile of the outcome distribution do and the grey area, the respective 95% confidence interval. The dotted straight line indicates the value at which the coefficient is not significant (equal to 0)

Figure 3 shows coefficients for the association between race/skin colour and the different quantiles in the distribution of number of morbidities, estimated via quantile model and adjusted for sex and age. The results show that the disparities between mixed-race and white participants differ as of the 22^nd^ quantile, with the least difference occurring in quantile 22.5, where mixed-race individuals have 0.12 (95% CI: 0.06–0.17) more morbidities than whites, rising to 0.27 (95% CI: 0.14–0.40) more in quantile 90 of the distribution. Comparing blacks and whites, the disparities start in the lower quantiles, but are sustained from the 20^th^ quantile upwards and increase, so that blacks had 0.11 (95% CI: 0.004–0.22) more morbidities than whites in quantile 7.5, rising to 0.47 (95% CI: 0.33–0.61) more in quantile 90.

In sensitivity analyses, adding in socioeconomic factors attenuated estimates in the age- and sex-adjusted models, but was insufficient to eliminate differences between blacks and whites in prevalence of multimorbidity (Additional File 4). Adding health risk behaviour to these models did not alter the estimates as compared with the models adjusted for socioeconomic factors only. The analyses considering the intersectional variable (Additional File 5) showed that the differences in prevalence (without adjustment) reside between men and women: the confidence intervals indicate overlapping between the estimates of white, mixed-race, and black men, and the prevalences of mixed-race and black women differ from white women. Age-adjusted models revealed that women showed a greater prevalence of multimorbidity than white men, especially black women, with a difference ranging from 20% (≥ 2 morbidities) to 201% (≥ 6 morbidities).

In the analysis with the outcome from a list of 13 morbidities (Additional File 6), the direction of the association was maintained, some differences ceased to exist, and other strong relations were maintained, e.g., when multimorbidity was defined as ≥ 6 morbidities, the prevalence was 89% greater among mixed-race individuals and 116% greater among blacks compared to whites. Lastly, in the analysis with the outcome from a list of six morbidities (Additional File 7), considering only those measured in ELSA-Brasil, the estimates of association were even higher for mixed-race and black participants concerning the original list of 16 morbidities. When multimorbidity was defined at the cutoff of ≥ 2 morbidities the adjusted estimate was 1.17 for mixed-race participants (95% CI: 1.13–1.20) and 1.27 for blacks (95% CI: 1.22–1.31). When was defined at the cutoff of ≥ 6 morbidities the estimate reached 2.73 for blacks (95% CI: 1.52–4.90).

## Discussion

This study with adults and older adults evidence of Brazil’s persistent racial inequalities in health and contributes to the understanding of the epidemiology of multimorbidity, especially in the context of low- and middle-income countries. Although multimorbidity is a common situation among ELSA participants, blacks displayed greater prevalence than whites and more morbidities in almost all quantiles of the distribution of the number of morbidities.

The prevalences found alert to the extent of multimorbidity in Brazil, which may be close to high-income countries with longer life expectancies, such as Portugal [42], Finland, Spain, and Poland [8]. Meanwhile, our prevalence was higher than those reported in analyses of the overall population of Latin America and the Caribbean, regarding both combined prevalence (43%; CI: 35%-51%) and combined prevalence excluding Brazilian studies (35%; CI: 26%-43%) [43].

The differences between the racial groups diverged from other Brazilian studies of the general population, which have reported greater prevalence in whites [22, 44], but these studies include adults 18 years of age and older, and diagnoses are self-reported. Rather, they approximate to those of the Longitudinal Study of the Health of Brazilian Older Adults, which specified multimorbidity as ≥ 2 and ≥ 3 morbidities, finding, respectively, 71.4% and 50.1% among blacks, 68.5% and 48.4% among whites, and 66.2% and 45.3% among mixed-race individuals [23].

Regarding the mean counts of morbidities, they were close to those reported in the Health and Retirement Study (USA), in which whites were found, on average, to live with 2.04 morbidities and blacks with 2.34 when follow up began [11], and, after adjustment, the count for blacks continued higher [10]. In ELSA, black and mixed-race participants returned higher counts in around 80% of the quantiles (particularly in the higher quantiles) of the distribution of number of morbidities after adjustment for age and sex. That evidence is an alert to the need to understand the mechanisms leading certain population groups to fall ill more than others.

Concerning the assessment of multimorbidity, the quantile analysis favours an approach reaching beyond the “average patient”. While the mean morbidity counts between racial groups were close (between 2.45 and 2.78 morbidities), and a linear or Poisson statistical model would work with these mean values, the quantile model illustrated that differences between racial groups widen as the number of coexisting morbidities is higher. For a population-based approach, it reveals the groups towards which health-promoting and disease-preventing interventions need to be directed so that the distribution curve of coexisting morbidities is shifted to the left.

Consistent with previous reports, an inverse relationship was found between prevalence and the cutoff used to specify multimorbidity [17, 45]. The traditional definition of multimorbidity (≥ 2 morbidities) revealed a high overall prevalence (70%) that was reduced to 46% when the cutoff changed to ≥ 3 morbidities. Concomitantly, the increase in the cutoff resulted in a greater estimate of association between race/skin colour and multimorbidity (especially for black participants). This evidence corroborates other discussions that suggest that increasing the cutoff in the operationalisation of multimorbidity may improve the specificity and provide greater differentiation between groups [17, 46]. For approaches at the individual level, it may identify those in a situation of complex multimorbidity, with multiple impaired body systems, which from a clinical point of view may be more meaningful to define patients who need differentiated integrated care.

Both the analysis by quantiles and the analysis with cutoff to define the multimorbidity led to the same conclusion: black individuals, especially, are in a situation of higher illness, with more coexisting morbidities. The quantile analysis is a resource when data about individuals are complete, and it is possible to define the exact number of morbidities. Alternatively, a cutoff for multimorbidity can be used in the presence of missing data when it is not possible to determine with accuracy the number of coexisting morbidities (e.g., when from a list of twenty morbidities there are records for only ten of them, but among these ten the individual has six).

Consistent with previous reports, our results suggest that the prevalence of multimorbidity is higher with advancing age and among women [3, 5, 6, 8]. The age acted as a negative confounder, underestimating the association between race/skin colour and multimorbidity when not controlled. Although the proportional distribution of racial groups by age group is close, the white group had 23.68% of individuals aged 60 years or more, against 17.34% of the mixed-race group and 19.6% of the black group. Since illness and diagnosis are related to advancing age, not controlling for age underestimated the true association. In addition, sex acted as a positive confounder in the association between black race/skin colour and multimorbidity (overestimating the association when not controlled). However, sex was a negative confounder in the association between mixed-race and multimorbidity. These effects are possibly explained by the proportion of women among the racial groups, since the black group presented a higher proportion of women (60.81%) in relation to the white group (53.76%), while the mixed-race group presented a lower proportion (51.62%).

This study found differences, by racial group, in the prevalences of multimorbidity and some morbidities (Additional File 3), complementing prior observations of racial inequalities in isolated diseases [47–49], but mixed-race and black women seem to be in worse health situations. That intersectionality (race and gender) can improve the documentation of health inequalities by addressing contextual factors that act jointly in producing illness [50]. The sensitivity analyses conducted here, considering an intersectional category for the independent variable, revealed no difference in multimorbidity in mixed-race and black men compared with white men, but rather that black women are in the worst situation (Additional File 5).

The results of this study should be read with certain points in mind. Adjustment for socioeconomic factors (Additional File 4) attenuated the magnitude of the association without fully explaining the associations found (especially for blacks). There are at least four reasons why race/skin colour still matters for health even when socioeconomic status is considered in analyses [34]. Firstly, health is affected by exposure to adversity throughout the life course [34]. Brazilian studies, population-based and nationwide, revealed that racial inequalities in health in the country are present from the beginning of life, e.g., with mixed-race and black women presenting a greater chance of inadequate prenatal care (which involves time to the start of prenatal care, number of appointments, exams, and receiving orientation), and with white children presenting better nutritional status in childhood [51, 52].

Secondly, there is a non-equivalence in the indicators of socioeconomic position across racial groups [34]. In our data, even though the mixed-race and black group has more than 50% of the individuals in the highest levels of education, they also have more than 50% of the individuals in the lowest levels of *per capita* family income. This is not the case for the white group. Thirdly, the most critical distinctive social exposure experienced by some racial groups is an additional effect of racism [34]. In ELSA-Brasil, black and mixed-race participants were more likely to live in economically segregated neighbourhoods [53], which may affect access to health-promoting conditions. Fourthly, some racial groups have an elevated risk of exposure to various psychosocial stressors [34], including experiences of discrimination throughout life – which were more frequently reported by mixed-race and black participants in ELSA-Brasil [54].

In addition, although Brazil does have a free, universal, public health system, a historical process of structural racism [21] has left substantial differences between racial groups in their access to health care and in care received [55, 56] which can have important implications for our results. The lack of diagnosis may mask even greater differences among the groups and, accordingly, our results may be underestimated, since ten of the 16 conditions on our list were self-reported. Corroborating this hypothesis, in the analysis considering six morbidities (Additional File 7), excluding the ten morbidities that are only self-reported in ELSA-Brasil, the differences between prevalences were amplified, and the association of mixed-race and black individuals with multimorbidity was stronger.

Another point relates to survival: life expectancy is shorter for blacks than for whites and levels of premature death (< 65 years) are higher [57, 58], so that blacks who reach advanced age may be less exposed to some risk factors throughout life and, as a result, display better state of health than the mean for their group or there may be differential survival among those with multimorbidity, which would be consistent with other situations of illness [59].

The data of this study offer no support for causal directionality, although it is reasonable to think that race/skin colour and accompanying experiences of racial discrimination precede the development of morbidities. Future studies are needed to investigate these factors’ roles in the prognosis and survival of different racial groups. The analysis of longitudinal data from ELSA-Brasil may answer whether the observed inequalities are related to disparities in the incidence and accumulation of morbidities, which have been documented regarding blacks in the USA [10, 11].

The patterns or severity of diseases or their impacts on daily and occupational activities or quality of life were not examined. Although the identification of which groups have the greatest number of morbidities is important to direct actions toward a target population, future studies are necessary to explore the differences in the situation of complex multimorbidity, in the identification of compromised body systems and in the pattern of diseases, which may differ between racial groups and differently impact the quality of life.

Lastly, generalisation of these findings to the overall population may be limited, because ELSA-Brasil included only civil servants in major cities, and black and mixed-race Brazilians historically have greater participation in informal occupations or are not occupied [60, 61]. For that reason, it is believed that racial inequalities in multimorbidity are even greater than this study has managed to measure.

The strengths of this study are that it used data on adults aged 35 and over who were neither hospitalised nor recruited by health services. Research into multimorbidity in younger adults deserves attention given the burden that multiple diseases place on the quality of life and health systems [3, 6, 13, 14]. In addition, it is essential to identify subgroups with more illness and vulnerable to multimorbidity, develop more equitable policies, and establish inter-sector strategies to act on the social determinants of health and mitigate their effects.

Another strength of this study is that the ELSA participants share certain occupational and socioeconomic similarities, and even after adjustment for variables of socioeconomic position and health risk behaviour (Additional File 4), differences in multimorbidity were attenuated, but not eliminated, especially for blacks, which is consistent with other reports [9, 33, 34]. Leading to the belief that the racial inequalities reported here reveal another facet of illness that is not captured by such indicators, but which may be life course-related in societies where unequal racial relations are determinants of illness.

## Conclusions

Although multimorbidity is a common situation among adults aged 35 to 74 years in a large Brazilian cohort, it affects different racial groups unequally, leaving blacks at the greatest disadvantage. Raising the cutoff point for defining multimorbidity revealed stronger associations between race/skin colour and multimorbidity, indicating not only higher prevalence of multimorbidity among mixed-race and black individuals than among whites, but that the former groups coexisted more often with more complex health situations (with more coexisting morbidities). The findings of this study suggest that using higher cutoff points to specify multimorbidity or analytical approaches that consider the number of coexisting morbidities, in addition to defining the presence or absence of the multimorbidity situation, can help identify high-risk groups. They also suggest that guidelines based on isolated diseases that do not address the social determinants of health may be incomplete and limited in understanding disease mechanisms and related therapeutic approaches. Intersectoral strategies are needed to address the underlying social causes of racial inequalities in health.

## Supplementary Information

**Additional file 1.** Flow diagram of exclusions in examining the association between race/skin colour and multimorbidity.

**Additionalfile 2.** Descriptive characteristics of participants with and without multimorbidity, for each multimorbidity cutoff.

**Additional file 3.** Prevalence of each morbidity by race/skin colour in specific age group and sex.

**Additional file 4.** Association between race/skin colour and multimorbidity adjusted for sociodemographic and behavioural factors.

**Additional file 5.** Association between the intersection of race/skin colour and gender and multimorbidity.

**Additional file 6.** Association between race/skin colour and multimorbidity by a list of 13 morbidities.

**Additionalfile 7.** Association between race/skin colour and multimorbidity by a list of six morbidities.

## Data Availability

The datasets used and/or analysed during the current study are available from the corresponding author on reasonable request.

## Abbreviations

AIC: Akaike Information Criterion
CI: Confidence interval
COPD: Chronic obstructive pulmonary disease
ELSA-Brasil: *Estudo Longitudinal de Saúde do Adulto*
IBGE: *Instituto Brasileiro de Geografia e Estatística*
PR: Prevalence ratio
USA: United States of America
