# Supplementary material for: Racial inequalities in multimorbidity: baseline of the Brazilian Longitudinal Study of Adult Health (ELSA-Brasil)
Source: BMC Public Health. 2022 Jul 9;22:1319. doi: 10.1186/s12889-022-13715-7 (PMC9270815; doi:10.1186/s12889-022-13715-7)

Additional File 1

Flow diagram of exclusions in examining the association between race/skin colour and multimorbidity, ELSA-Brasil baseline

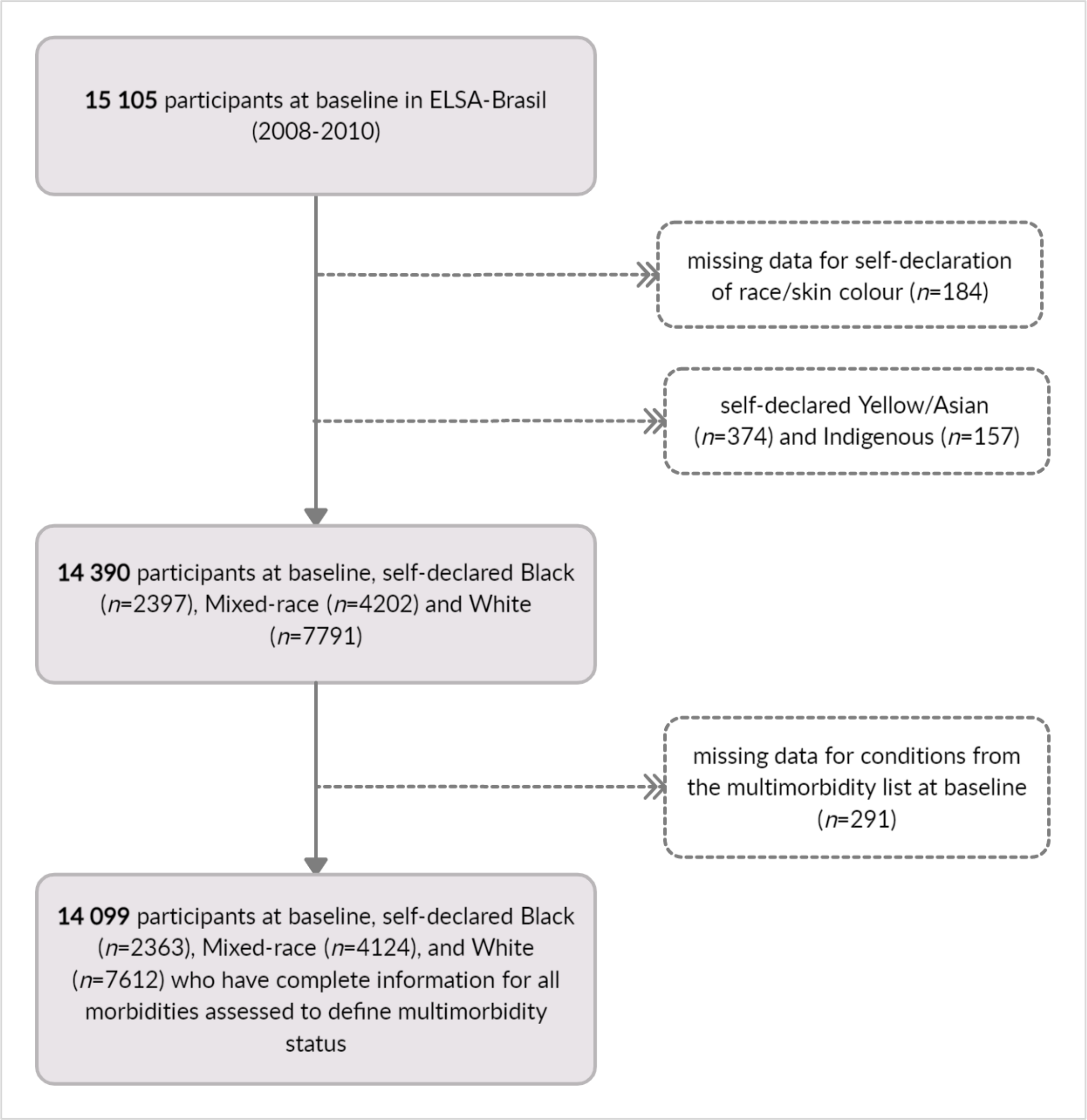

Supplement: Supplementary file 1 — Additional file 1. Flow diagram of exclusions in examining the association between race/skin colour and multimorbidity. [file 12889_2022_13715_MOESM1_ESM.pdf]
