# Supplementary material for: Racial inequalities in multimorbidity: baseline of the Brazilian Longitudinal Study of Adult Health (ELSA-Brasil)
Source: BMC Public Health. 2022 Jul 9;22:1319. doi: 10.1186/s12889-022-13715-7 (PMC9270815; doi:10.1186/s12889-022-13715-7)
Supplement: Supplementary file 2 — Additionalfile 2. Descriptive characteristics of participants with and without multimorbidity, for each multimorbidity cutoff. [file 12889_2022_13715_MOESM2_ESM.pdf]

Additional File 2

Descriptive characteristics of participants with and without multimorbidity (cutoff ≥2 morbidities), ELSA-Brasil baseline

Baseline characteristics[a]	Multimorbidity (cutoff ≥ 2 morbidities)[b]		*p* Value[c]
	No (%)	Yes (%)	
**Demographic**			
Age in years (n=14 099)			
Mean (standard deviation)	49.18 (8.51)	53.17 (9.02)	<0.001
Median (1st quartile - 3rd quartile)	48 (43-55)	53 (46-59)	
Sex (n=14 099)			
Male	2214 (52.50)	4227 (42.77)	<0.001
Female	2003 (47.50)	5655 (57.23)	
Race/skin colour (n=14 099)			
White	2409 (57.13)	5203 (52.65)	<0.001
Mixed-race	1210 (28.69)	2914 (29.49)	
Black	598 (14.18)	1765 (17.86)	
**Socioeconomic position**			
Education levels (n=14 099)			
Complete higher education	2384 (56.53)	4997 (50.57)	<0.001
Complete high school	1430 (33.91)	3519 (35.61)	
Complete elementary school	233 (5.53)	729 (7.38)	
Up to incomplete elementary school	170 (4.03)	637 (6.45)	
Per capita family income (n=14 059)			
5th (> US$ 1315.66 and ≤ US$ 3946.99)	682 (16.24)	1596 (16.19)	0.001
4th (> US$ 882.88 and ≤ US$ 1315.66)	1030 (24.52)	2146 (21.77)	
3rd (> US$ 519.25 and ≤ US$ 882.88)	870 (20.71)	2037 (20.66)	
2nd (> US$ 311.58 and ≤ US$ 519.25)	836 (19.90)	2024 (20.53)	
1st (≤ US$ 311.58)	782 (18.62)	2056 (20.85)	
Health insurance plans (n=14 098)			
Yes	2822 (66.92)	6807 (68.89)	0.022
No	1395 (33.08)	3074 (31.11)	
**Behaviours**			
Smoking (n=14 098)			
Non-smoker	2621 (62.15)	5423 (54.88)	<0.001
Former smoker	1057 (25.07)	3157 (31.95)	
Current smoker	539 (12.78)	1301 (13.17)	
Hazardous drinking (n=14 090)			
No	3911 (92.81)	9121 (92.36)	0.367
Yes	303 (7.19)	755 (7.64)	
Physical activity (n=13 899)			
Weak	3019 (72.78)	7661 (78.57)	<0.001
Moderate	715 (17.24)	1517 (15.56)	
Vigorous	414 (9.98)	573 (5.88)	

Notes: [a]The total number of participants with complete information for each of the characteristics is indicated in parentheses. [b]Percentages within each group with or without multimorbidity (sum 100% in the column), except for the numerical variable age for which mean, standard deviation, median, 1st and 3rd quartile are given. [c]Refers to the X^2 test for difference in percentages and Kruskal-Wallis test for age between groups with and without multimorbidity.

Descriptive characteristics of participants with and without multimorbidity (cutoff ≥3 morbidities), ELSA-Brasil baseline

Baseline characteristics[a]	Multimorbidity (cutoff ≥ 3 morbidities)[b]		*p* Value[c]
	No (%)	Yes (%)	
**Demographic**			
Age in years (n=14 099)			
Mean (standard deviation)	50.17 (8.71)	54.10 (9.01)	<0.001
Median (1st quartile - 3rd quartile)	49 (44-56)	54 (47-60)	
Sex (n=14 099)			
Male	2852 (50.55)	2589 (39.96)	<0.001
Female	3768 (49.45)	3890 (60.04)	
Race/skin colour (n=14 099)			
White	4254 (55.83)	3358 (51.83)	<0.001
Mixed-race	2233 (29.30)	1891 (29.19)	
Black	1133 (14.87)	1230 (18.98)	
**Socioeconomic position**			
Education levels (n=14 099)			
Complete higher education	4275 (56.10)	3106 (47.94)	<0.001
Complete high school	2548 (33.44)	2401 (37.06)	
Complete elementary school	450 (5.91)	512 (7.90)	
Up to incomplete elementary school	347 (4.55)	460 (7.10)	
Per capita family income (n=14 059)			
5th (> US$ 1315.66 and ≤ US$ 3946.99)	1243 (16.37)	1035 (16.01)	<0.001
4th (> US$ 882.88 and ≤ US$ 1315.66)	1803 (23.75)	1373 (21.23)	
3rd (> US$ 519.25 and ≤ US$ 882.88)	1619 (21.32)	1288 (19.92)	
2nd (> US$ 311.58 and ≤ US$ 519.25)	1495 (19.69)	1365 (21.11)	
1st (≤ US$ 311.58)	1433 (18.87)	1405 (21.73)	
Health insurance plans (n=14 098)			
Yes	5191 (68.13)	4438 (68.50)	0.655
No	2428 (31.87)	2041 (31.50)	
**Behaviours**			
Smoking (n=14 098)			
Non-smoker	4578 (60.08)	3466 (53.50)	<0.001
Former smoker	2032 (26.67)	2182 (33.68)	
Current smoker	1010 (13.25)	830 (12.81)	
Hazardous drinking (n=14 090)			
No	7058 (92.66)	5974 (92.29)	0.424
Yes	559 (7.34)	499 (7.71)	
Physical activity (n=13 899)			
Weak	5530 (73.70)	5150 (80.52)	<0.001
Moderate	1273 (16.97)	959 (14.99)	
Vigorous	700 (9.33)	287 (4.49)	

Notes: [a]The total number of participants with complete information for each of the characteristics is indicated in parentheses. [b]Percentages within each group with or without multimorbidity (sum 100% in the column), except for the numerical variable age for which mean, standard deviation, median, 1st and 3rd quartile are given. [c]Refers to the X^2 test for difference in percentages and Kruskal-Wallis test for age between groups with and without multimorbidity.

Descriptive characteristics of participants with and without multimorbidity (cutoff ≥4 morbidities), ELSA-Brasil baseline

**Baseline characteristics**[a]	**Multimorbidity (cutoff ≥ 4 morbidities)**[b]		***p* Value**[c]
	**No (%)**	**Yes (%)**	
**Demographic**			
Age in years (n=14 099)			
Mean (standard deviation)	50.90 (8.84)	55.10 (8.96)	<0.001
Median (1st quartile - 3rd quartile)	50 (44-57)	55 (48-61)	
Sex (n=14 099)			
Male	5135 (48.98)	1306 (36.13)	<0.001
Female	5349 (51.02)	2309 (63.87)	
Race/skin colour (n=14 099)			
White	5789 (55.22)	1823 (50.43)	<0.001
Mixed-race	3075 (29.33)	1049 (29.02)	
Black	1620 (15.45)	743 (20.55)	
**Socioeconomic position**			
Education levels (n=14 099)			
Complete higher education	5733 (54.68)	1648 (45.59)	<0.001
Complete high school	3565 (34.00)	1384 (38.28)	
Complete elementary school	673 (6.42)	289 (7.99)	
Up to incomplete elementary school	513 (4.89)	294 (8.13)	
Per capita family income (n=14 059)			
5th (> US$ 1315.66 and ≤ US$ 3946.99)	1712 (16.38)	566 (15.70)	<0.001
4th (> US$ 882.88 and ≤ US$ 1315.66)	2418 (23.13)	758 (21.02)	
3rd (> US$ 519.25 and ≤ US$ 882.88)	2220 (21.24)	687 (19.05)	
2nd (> US$ 311.58 and ≤ US$ 519.25)	2101 (20.10)	759 (21.05)	
1st (≤ US$ 311.58)	2002 (19.15)	836 (23.18)	
Health insurance plans (n=14 098)			
Yes	7143 (68.14)	2486 (68.77)	0.496
No	3340 (31.86)	1129 (31.23)	
**Behaviours**			
Smoking (n=14 098)			
Non-smoker	6116 (58.34)	1928 (53.35)	<0.001
Former smoker	2960 (28.23)	1254 (34.70)	
Current smoker	1408 (13.43)	432 (11.95)	
Hazardous drinking (n=14 090)			
No	9692 (92.49)	3340 (92.50)	0.999
Yes	787 (7.51)	271 (7.50)	
Physical activity (n=13 899)			
Weak	7763 (75.19)	2917 (81.62)	<0.001
Moderate	1717 (16.63)	515 (14.41)	
Vigorous	845 (8.18)	142 (3.97)	

Notes: [a]The total number of participants with complete information for each of the characteristics is indicated in parentheses. [b]Percentages within each group with or without multimorbidity (sum 100% in the column), except for the numerical variable age for which mean, standard deviation, median, 1st and 3rd quartile are given. [c]Refers to the X^2 test for difference in percentages and Kruskal-Wallis test for age between groups with and without multimorbidity.

Descriptive characteristics of participants with and without multimorbidity (cutoff ≥5 morbidities), ELSA-Brasil baseline

**Baseline characteristics**[a]	**Multimorbidity (cutoff ≥ 5 morbidities)**[b]		***p* Value**[c]
	**No (%)**	**Yes (%)**	
**Demographic**			
Age in years (n=14 099)			
Mean (standard deviation)	51.38 (8.93)	56.20 (8.85)	<0.001
Median (1st quartile - 3rd quartile)	50 (45-57)	56 (50-62)	
Sex (n=14 099)			
Male	5854 (47.36)	587 (33.76)	<0.001
Female	6506 (52.64)	1152 (66.24)	
Race/skin colour (n=14 099)			
White	6742 (54.55)	870 (50.03)	<0.001
Mixed-race	3626 (29.34)	498 (28.64)	
Black	1992 (16.12)	371 (21.33)	
**Socioeconomic position**			
Education levels (n=14 099)			
Complete higher education	6634 (53.67)	747 (42.96)	<0.001
Complete high school	4279 (34.62)	670 (38.53)	
Complete elementary school	791 (6.40)	171 (9.83)	
Up to incomplete elementary school	656 (5.31)	151 (8.68)	
Per capita family income (n=14 059)			
5th (> US$ 1315.66 and ≤ US$ 3946.99)	2002 (16.25)	276 (15.90)	<0.001
4th (> US$ 882.88 and ≤ US$ 1315.66)	2817 (22.86)	359 (20.68)	
3rd (> US$ 519.25 and ≤ US$ 882.88)	2592 (21.03)	315 (18.15)	
2nd (> US$ 311.58 and ≤ US$ 519.25)	2485 (20.17)	375 (21.60)	
1st (≤ US$ 311.58)	2427 (19.69)	411 (23.68)	
Health insurance plans (n=14 098)			
Yes	8417 (68.10)	1212 (69.70)	0.191
No	3942 (31.90)	527 (30.30)	
**Behaviours**			
Smoking (n=14 098)			
Non-smoker	7141 (57.78)	903 (51.96)	<0.001
Former smoker	3568 (28.87)	646 (37.17)	
Current smoker	1651 (13.36)	189 (10.87)	
Hazardous drinking (n=14 090)			
No	11413 (92.38)	1619 (93.31)	0.180
Yes	942 (7.62)	116 (6.69)	
Physical activity (n=13 899)			
Weak	9269 (76.13)	1411 (81.89)	<0.001
Moderate	1980 (16.26)	252 (14.63)	
Vigorous	927 (7.61)	60 (3.48)	

Notes: [a]The total number of participants with complete information for each of the characteristics is indicated in parentheses. [b]Percentages within each group with or without multimorbidity (sum 100% in the column), except for the numerical variable age for which mean, standard deviation, median, 1st and 3rd quartile are given. [c]Refers to the X^2 test for difference in percentages and Kruskal-Wallis test for age between groups with and without multimorbidity.

Descriptive characteristics of participants with and without multimorbidity (cutoff ≥6 morbidities), ELSA-Brasil baseline

Baseline characteristics[a]	Multimorbidity (cutoff ≥ 6 morbidities)[b]		*p* Value[c]
	No (%)	Yes (%)	
**Demographic**			
Age in years (n=14 099)			
Mean (standard deviation)	51.72 (9.01)	56.72 (8.70)	<0.001
Median (1st quartile - 3rd quartile)	51 (45-58)	57 (51-63)	
Sex (n=14 099)			
Male	6233 (46.66)	208 (28.11)	<0.001
Female	7126 (53.34)	532 (71.89)	
Race/skin colour (n=14 099)			
White	7255 (54.31)	357 (48.24)	<0.001
Mixed-race	3902 (29.21)	222 (30.00)	
Black	2202 (16.48)	161 (21.76)	
**Socioeconomic position**			
Education levels (n=14 099)			
Complete higher education	7078 (52.98)	303 (40.95)	<0.001
Complete high school	4669 (34.95)	280 (37.84)	
Complete elementary school	882 (6.60)	80 (10.81)	
Up to incomplete elementary school	730 (5.46)	77 (10.41)	
Per capita family income (n=14 059)			
5th (> US$ 1315.66 and ≤ US$ 3946.99)	2171 (16.30)	107 (14.48)	<0.001
4th (> US$ 882.88 and ≤ US$ 1315.66)	3035 (22.79)	141 (19.08)	
3rd (> US$ 519.25 and ≤ US$ 882.88)	2774 (20.83)	133 (18.00)	
2nd (> US$ 311.58 and ≤ US$ 519.25)	2697 (20.25)	163 (22.06)	
1st (≤ US$ 311.58)	2643 (19.84)	195 (26.39)	
Health insurance plans (n=14 098)			
Yes	9115 (68.24)	514 (69.46)	0.512
No	4243 (31.76)	226 (30.54)	
**Behaviours**			
Smoking (n=14 098)			
Non-smoker	7657 (57.32)	387 (52.30)	0.005
Former smoker	3954 (29.60)	260 (35.14)	
Current smoker	1747 (13.08)	93 (12.57)	
Hazardous drinking (n=14 090)			
No	12 337 (92.40)	695 (94.17)	0.087
Yes	1015 (7.60)	43 (5.83)	
Physical activity (n=13 899)			
Weak	10 071 (76.51)	609 (82.74)	<0.001
Moderate	2126 (16.15)	106 (14.40)	
Vigorous	966 (7.34)	21 (2.85)	

Notes: [a]The total number of participants with complete information for each of the characteristics is indicated in parentheses. [b]Percentages within each group with or without multimorbidity (sum 100% in the column), except for the numerical variable age for which mean, standard deviation, median, 1st and 3rd quartile are given. [c]Refers to the X^2 test for difference in percentages and Kruskal-Wallis test for age between groups with and without multimorbidity.
