# Supplementary material for: Racial inequalities in multimorbidity: baseline of the Brazilian Longitudinal Study of Adult Health (ELSA-Brasil)
Source: BMC Public Health. 2022 Jul 9;22:1319. doi: 10.1186/s12889-022-13715-7 (PMC9270815; doi:10.1186/s12889-022-13715-7)
Supplement: Supplementary file 3 — Additional file 3. Prevalence of each morbidity by race/skin colour in specific age group and sex. [file 12889_2022_13715_MOESM3_ESM.pdf]

Additional File 3

Prevalence of each morbidity by race/skin colour in specific age group and sex, ELSA-Brasil baseline

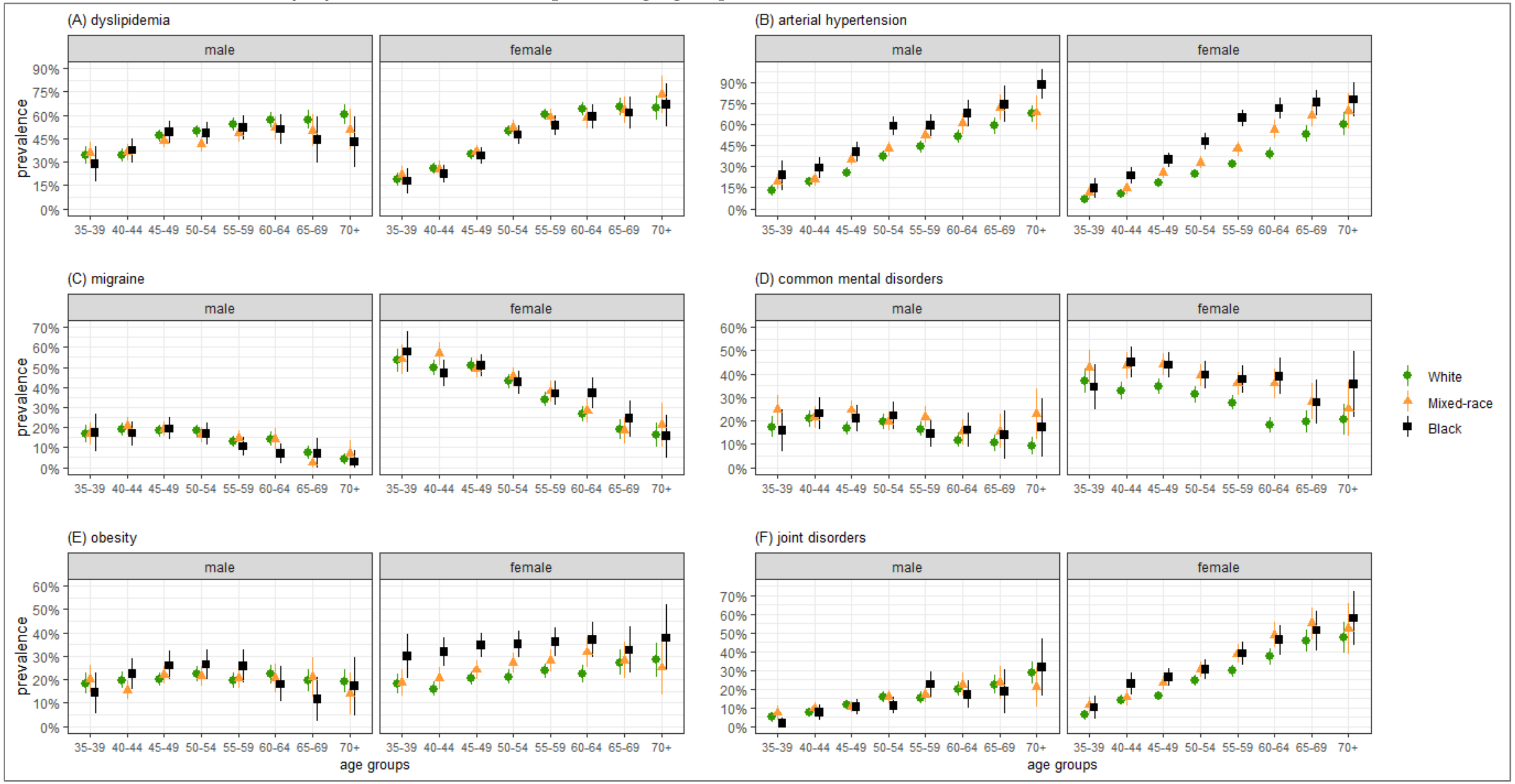

Notes: vertical lines correspond to 95% confidence interval of the prevalence estimates.

Prevalence of each morbidity by race/skin colour in specific age group and sex, ELSA-Brasil baseline

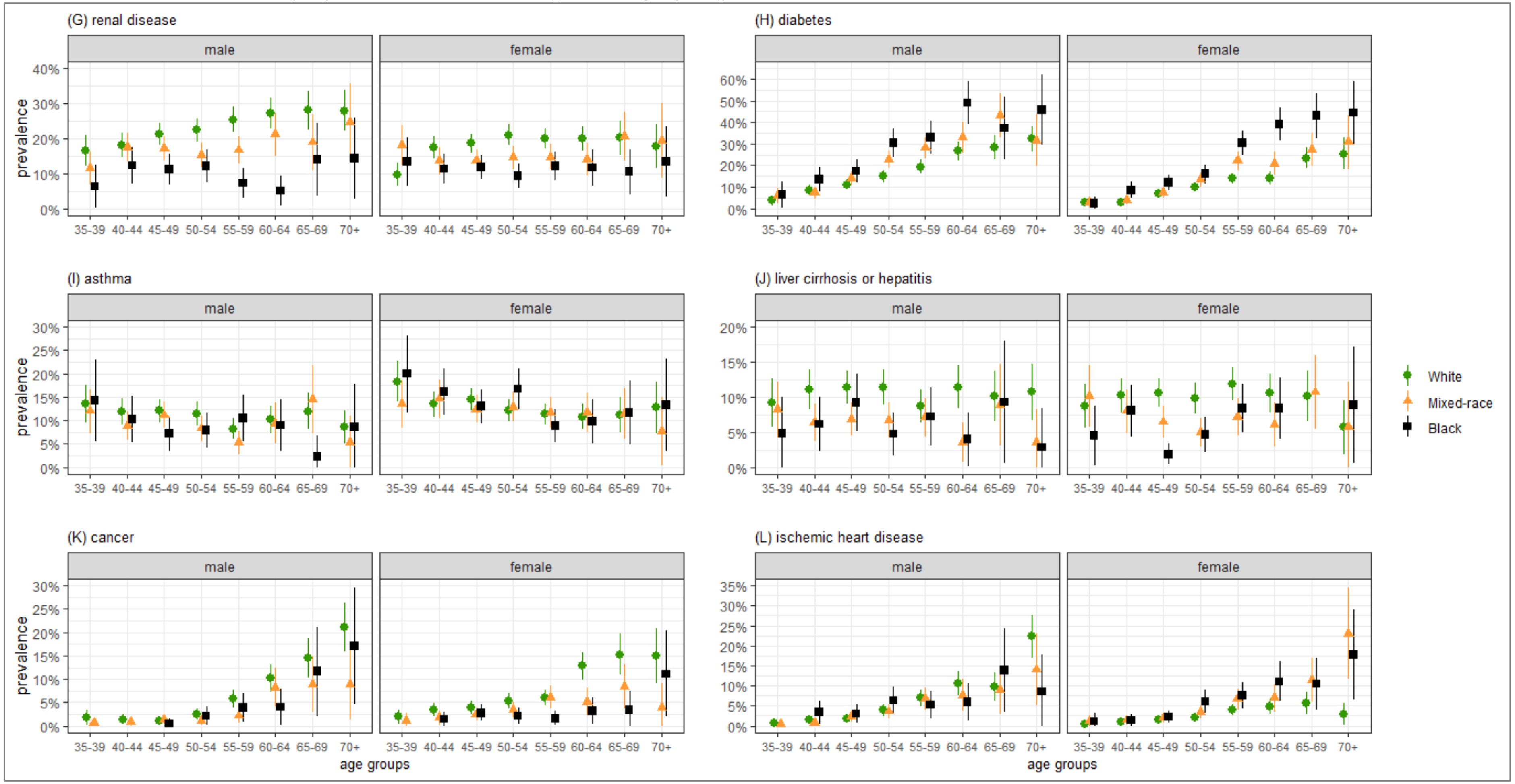

Notes: the vertical lines correspond to the 95% confidence interval of the prevalence estimates.

Prevalence of each morbidity by race/skin colour in specific age group and sex, ELSA-Brasil baseline

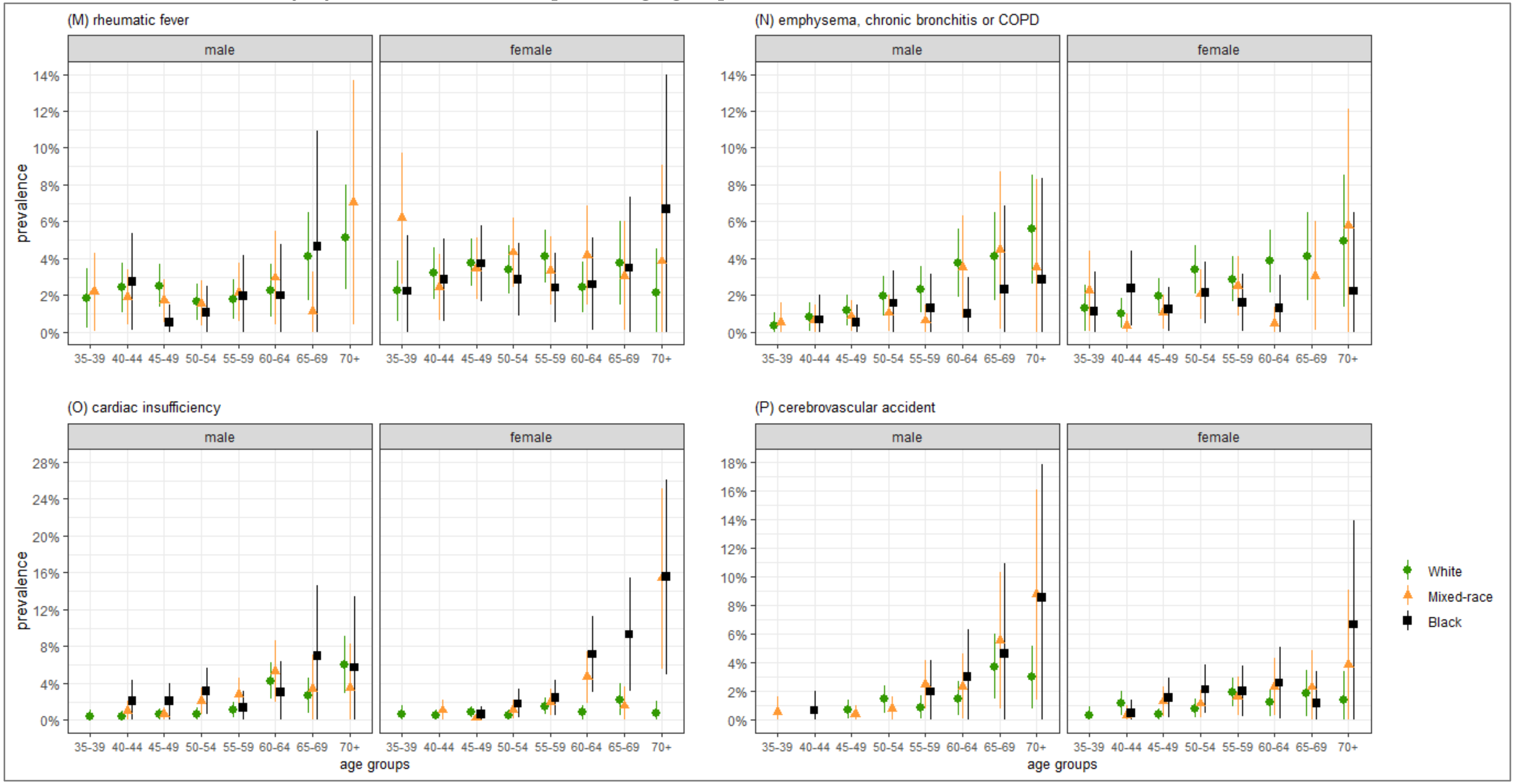

Notes: the vertical lines correspond to the 95% confidence interval of the prevalence estimates.
