# Supplementary material for: Racial inequalities in multimorbidity: baseline of the Brazilian Longitudinal Study of Adult Health (ELSA-Brasil)
Source: BMC Public Health. 2022 Jul 9;22:1319. doi: 10.1186/s12889-022-13715-7 (PMC9270815; doi:10.1186/s12889-022-13715-7)
Supplement: Supplementary file 4 — Additional file 4. Association between race/skin colour and multimorbidity adjusted for sociodemographic and behavioural factors. [file 12889_2022_13715_MOESM4_ESM.pdf]

Additional File 4

Association between race/skin colour and multimorbidity adjusted for sociodemographic and behavioural factors, baseline ELSA-Brasil

Multimorbidity cutoff[a]	Model 1[b]	Model 2[c]
	**PR (95% CI)**	**PR (95% CI)**
**≥ 2 morbidities**		
Mixed-race	1.03 (1.01-1.06)*	1.03 (1.01-1.06)*
Black	1.06 (1.03-1.09)***	1.06 (1.03-1.10)***
AIC	*16 363*	*16 043*
**≥ 3 morbidities**		
Mixed-race	1.02 (0.98-1.07)	1.02 (0.98-1.07)
Black	1.10 (1.05-1.16)***	1.10 (1.05-1.16)***
AIC	*18 442*	*18 039*
**≥ 4 morbidities**		
Mixed-race	1.02 (0.95-1.10)	1.02 (0.95-1.10)
Black	1.18 (1.08-1.28)***	1.18 (1.08-1.28)***
AIC	*15 109*	*14 836*
**≥ 5 morbidities**		
Mixed-race	0.98 (0.88-1.10)	0.99 (0.88-1.11)
Black	1.15 (1.01-1.31)*	1.15 (1.01-1.32)*
AIC	*9878*	*9714*
**≥ 6 morbidities**		
Mixed-race	1.01 (0.84-1.21)	1.02 (0.85-1.22)
Black	1.10 (0.90-1.35)	1.11 (0.90-1.36)
AIC	*5428*	*5376*

Notes: PR= prevalence ratios; 95% CI= 95% confidence interval; AIC= Akaike Information Criterion. [a]Reference category in all models: white race/skin colour. [b]Model 1: adjusted for age, sex, education levels, monthly per capita family income and health insurance plans. [c]Model 2: adjusted for age, sex, education levels, monthly per capita family income, health insurance plans, smoking and physical activity. Model 2 for multimorbidity specified as ≥ 3 morbidities also included adjustment for hazardous drinking, which was significant in this model only. Significance: *** p value ≤ 0.001; ** 0.001 < p value ≤ 0.01; * 0.01 < p value < 0.05.
