# Supplementary material for: Racial inequalities in multimorbidity: baseline of the Brazilian Longitudinal Study of Adult Health (ELSA-Brasil)
Source: BMC Public Health. 2022 Jul 9;22:1319. doi: 10.1186/s12889-022-13715-7 (PMC9270815; doi:10.1186/s12889-022-13715-7)
Supplement: Supplementary file 5 — Additional file 5. Association between the intersection of race/skin colour and gender and multimorbidity. [file 12889_2022_13715_MOESM5_ESM.pdf]

Additional File 5

Association between the intersection of race/skin colour and gender and multimorbidity, ELSA-Brasil baseline

Multimorbidity cutoff[a]	Prevalence of multimorbidity (95% CI)	PR (95% CI)	
		Crude (95% CI)	Adjusted for age
**≥ 2 morbidities**			
White men	65.94 (64.36-67.49)	1.00 (reference)	1.00 (reference)
Mixed-race men	64.46 (62.35-66.53)	0.98 (0.95-1.02)	1.01 (0.98-1.05)
Black men	66.95 (63.86-69.91)	1.01 (0.97-1.06)	1.03 (0.99-1.07)
White women	70.43 (69.01-71.81)	1.06 (1.03-1.09)***	1.07 (1.04-1.10)***
Mixed-race women	76.47 (74.62-78.22)	1.15 (1.11-1.78)***	1.16 (1.13-1.19)***
Black women	79.68 (77.52-81.68)	1.19 (1.15-1.22)***	1.20 (1.16-1.23)***
AIC		*17 042*	*16 437*
**≥ 3 morbidities**			
White men	40.74 (39.13-42.37)	1.00 (reference)	1.00 (reference)
Mixed-race men	38.25 (36.14-40.40)	0.94 (0.89-1.01)	1.00 (0.94-1.07)
Black men	42.33 (39.19-45.54)	1.04 (0.96-1.12)	1.08 (1.00-1.16)
White women	47.02 (45.49-48.55)	1.14 (1.09-1.20)***	1.18 (1.12-1.24)***
Mixed-race women	52.98 (50.86-55.10)	1.28 (1.22-1.34)***	1.34 (1.27-1.40)***
Black women	58.32 (55.75-60.84)	1.40 (1.33-1.47)***	1.45 (1.38-1.52)***
AIC		*19 241*	*18 557*
**≥ 4 morbidities**			
White men	20.74 (19.43-22.11)	1.00 (reference)	1.00 (reference)
Mixed-race men	18.85 (17.19-20.62)	0.92 (0.82-1.02)	1.01 (0.90-1.12)
Black men	21.60 (19.07-24.36)	1.04 (0.91-1.18)	1.11 (0.97-1.26)
White women	26.71 (25.38-28.09)	1.28 (1.18-1.38)***	1.34 (1.24-1.46)***
Mixed-race women	31.61 (29.67-33.62)	1.50 (1.38-1.62)***	1.62 (1.49-1.76)***
Black women	37.79 (35.32-40.32)	1.77 (1.63-1.92)***	1.91 (1.75-2.07)***
AIC		*15 815*	*15 218*
**≥ 5 morbidities**			
White men	9.89 (8.94-10.92)	1.00 (reference)	1.00 (reference)
Mixed-race men	7.57 (6.49-8-81)	0.77 (0.64-0.92)	0.88 (0.73-1.06)
Black men	9.50 (7.78-11.56)	0.96 (0.77-1.20)	1.05 (0.84-1.31)
White women	12.76 (11.77-13.81)	1.29 (1.14-1.46)***	1.38 (1.21-1.57)***
Mixed-race women	16.30 (14.79-17.93)	1.64 (1.43-1.87)***	1.83 (1.60-2.10)***
Black women	19.69 (17.72-21.83)	1.97 (1.72-2.25)***	2.18 (1.90-2.51)***
AIC		*10 377*	*9944*
**≥ 6 morbidities**			
White men	3.58 (3.02-4.25)	1.00 (reference)	1.00 (reference)
Mixed-race men	3.06 (2.39-3.91)	0.86 (0.64-1.15)	1.00 (0.74-1.35)
Black men	2.27 (1.49-3.44)	0.64 (0.41-1.001)*	0.70 (0.45-1.11)
White women	5.65 (4.98-6.40)	1.57 (1.28-1.94)***	1.70 (1.38-2.10)***
Mixed-race women	7.56 (6.51-8.76)	2.10 (1.68-2.61)***	2.38 (1.90-2.98)***
Black women	9.74 (8.32-11.39)	2.69 (2.15-3.36)***	3.01 (2.40-3.78)***
AIC		*5680*	*5464*

Notes: PR= prevalence ratios; 95% CI= 95% confidence interval; AIC= Akaike Information Criterion. [a]The total participants in each category is: 3520 (White men), 1995 (Mixed-race men), 926 (Black men), 4092 (White women), 2129 (Mixed-race women) and 1437 (Black women). Significance: *** p value ≤ 0.001; ** 0.001 < p value ≤ 0.01; * 0.01 < p value < 0.05.
