# Supplementary material for: Racial inequalities in multimorbidity: baseline of the Brazilian Longitudinal Study of Adult Health (ELSA-Brasil)
Source: BMC Public Health. 2022 Jul 9;22:1319. doi: 10.1186/s12889-022-13715-7 (PMC9270815; doi:10.1186/s12889-022-13715-7)
Supplement: Supplementary file 6 — Additional file 6. Association between race/skin colour and multimorbidity by a list of 13 morbidities. [file 12889_2022_13715_MOESM6_ESM.pdf]

Additional File 6

Association between race/skin colour and multimorbidity by a list of 13 morbidities, ELSA-Brasil baseline

**Multimorbidity cutoff**[a]	**Crude Estimate**	**Adjusted for age and sex**
	**PR (95% CI)**	**PR (95% CI)**
**≥ 2 morbidities (43.15%)**[b]		
Mixed-race	1.01 (0.97-1.05)	1.03 (0.99-1.08)
Black	1.07 (1.02-1.13)*	1.05 (1.00-1.11)
AIC	*19 279*	*18 753*
**≥ 3 morbidities (19.44%)**[b]		
Mixed-race	1.01 (0.94-1.09)	1.06 (0.98-1.14)
Black	1.12 (1.02-1.23)*	1.10 (1.00-1.20)
AIC	*13 889*	*13 441*
**≥ 4 morbidities (7.35%)**[b]		
Mixed-race	1.17 (1.02-1.34)*	1.25 (1.09-1.44)**
Black	1.28 (1.10-1.50)**	1.25 (1.07-1.47)**
AIC	*7398*	*7073*
**≥ 5 morbidities (2.27%)**[b]		
Mixed-race	1.12 (0.88-1.44)	1.22 (0.95-1.56)
Black	1.15 (0.85-1.54)	1.12 (0.83-1.51)
AIC	*3060*	*2928*
**≥ 6 morbidities (0.68%)**[b]		
Mixed-race	1.70 (1.07-2.70)*	1.89 (1.18-3.01)**
Black	2.18 (1.31-3.60)**	2.16 (1.30-3.59)**
AIC	*1145*	*1091*

Notes: PR= prevalence ratios; 95% CI= 95% confidence interval; AIC= Akaike Information Criterion. List of 13 morbidities: cancer; rheumatic fever; ischemic heart disease; cardiac insufficiency; cerebrovascular accident; emphysema, chronic bronchitis or chronic obstructive pulmonary disease; asthma; liver cirrhosis or hepatitis; joint disorders; renal disease; diabetes; common non-psychotic mental disorders; and migraine. [a]Reference category in all models: white race/skin colour. [b]Proportion in brackets indicates prevalence of multimorbidity at the corresponding cutoff. Significance: *** p value ≤ 0.001; ** 0.001 < p value ≤ 0.01; * 0.01 < p value < 0.05.
