# Supplementary material for: Racial inequalities in multimorbidity: baseline of the Brazilian Longitudinal Study of Adult Health (ELSA-Brasil)
Source: BMC Public Health. 2022 Jul 9;22:1319. doi: 10.1186/s12889-022-13715-7 (PMC9270815; doi:10.1186/s12889-022-13715-7)
Supplement: Supplementary file 7 — Additionalfile 7. Association between race/skin colour and multimorbidity by a list of six morbidities. [file 12889_2022_13715_MOESM7_ESM.pdf]

Additional File 7

Association between race/skin colour and multimorbidity by a list of 6 morbidities, ELSA-Brasil baseline

Multimorbidity cutoff[a]	Crude Estimate	Adjusted for age and sex
	PR (95% CI)	PR (95% CI)
**≥ 2 morbidities (54.23%)[b]**		
Mixed-race	1.13 (1.09-1.17)***	1.17 (1.13-1.20)***
Black	1.26 (1.22-1.31)***	1.27 (1.22-1.31)***
AIC	*19 297*	*18 756*
**≥ 3 morbidities (26.49%)[b]**		
Mixed-race	1.23 (1.16-1.31)***	1.30 (1.22-1.39)***
Black	1.60 (1.50-1.71)***	1.63 (1.53-1.75)***
AIC	*16 218*	*15 646*
**≥ 4 morbidities (9.66%)[b]**		
Mixed-race	1.31 (1.16-1.47)***	1.39 (1.24-1.57)***
Black	2.00 (1.78-2.26)***	2.02 (1.79-2.29)***
AIC	*8844*	*8590*
**≥ 5 morbidities (2.51%)[b]**		
Mixed-race	1.48 (1.15-1.89)**	1.57 (1.22-2.01)***
Black	2.57 (2.01-3.29)***	2.52 (1.96-3.23)***
AIC	*3261*	*3179*
**≥ 6 morbidities (0.42%)[b]**		
Mixed-race	1.08 (0.56-2.08)	1.13 (0.59-2.19)
Black	2.82 (1.57-5.05)***	2.73 (1.52-4.90)***
AIC	*758*	*748*

Notes: PR= prevalence ratios; 95% CI= 95% confidence interval; AIC= Akaike Information Criterion. List of 6 morbidities: dyslipidaemia; arterial hypertension; migraine; common mental disorders; obesity; and diabetes. [a]Reference category in all models: white race/skin colour. [b]Proportion in brackets indicates prevalence of multimorbidity at the corresponding cutoff. Significance: *** p value ≤ 0.001; ** 0.001 < p value ≤ 0.01; * 0.01 < p value < 0.05.
